# CSEC**^+^** framework assessment dataset: Expert evaluations of cybersecurity skills for job profiles in Europe

**DOI:** 10.1016/j.dib.2023.109285

**Published:** 2023-05-29

**Authors:** Carlos E. Budde, Anni Karinsalo, Silvia Vidor, Jarno Salonen, Fabio Massacci

**Affiliations:** a University of Trento, Trento I-38122, Italy; b VTT Technical Research Centre of Finland, Oulu FI-90571, Finland; c VTT Technical Research Centre of Finland, Tampere FI-33101, Finland; d Vrije Universiteit Amsterdam, Amsterdam, 1081 HV, the Netherlands

**Keywords:** Cybersecurity, Professional skills, Assessment framework, Human security, Education

## Abstract

This dataset contains expert assessments of the cybersecurity skills required for six job profiles in Europe, as determined via surveys responded by cybersecurity experts from academia and industry. The data can be used to identify educational needs in the cybersecurity sector and compare against other frameworks.

The six cybersecurity-oriented job profiles used in the surveys are: General cybersec auditor; Technical cybersec auditor; Threat modelling engineer; Security engineer; Enterprise cybersecurity practitioner; Cybersecurity analyst. Data—i.e. expert assessments—was collected via surveys, targeted at European experts in cybersecurity from academia and industry. Respondents characterised the skills needed to perform in six job profiles using the CSEC**^+^** framework: a cybersecurity skills framework prepared as a spreadsheet where cybersecurity skills must be ranked in a Likert scale from 0 (*irrelevant*) to 4 (*advance knowledge needed*). Metadata requested included the type of organisation of the respondent (*Large company, SME, Academic/Research, Public administration, Other*) and the country of origin.

There were three data-collection phases: (1) an initial phase, used also to refine later larger-scale processes, carried out in Oct 2021–Jan 2022 and resulting in 13 expert assessments from four EU countries; (2) a second phase implemented as an online service broadcast to a larger audience, carried out in Mar–Apr 2022 and resulting in 15 assessments from eight European countries; (3) and a third phase, allowing direct online input and distributed in PC and mobile form, carried out in Sep–Oct 2022 and resulting in 32 assessments from ten European countries.

The raw data gathered was stored and processed via spreadsheets, computing statistical information (mean, stdev) on how much each cybersecurity skill and area was deemed necessary to perform in each job profile. This is visualised as a heatmap where colour intensity symbolises value, and circle diffusion symbolises spread. Processed data further includes visualisations on how the area of origin of the respondent (academia, as in “producer of education”, vs. industry, as in “consumer of education”) influences the responses. This is shown as bar plots, where whiskers represent confidence intervals used for statistical-significance tests.

This data can serve as basis to understand the educational needs for the cybersecurity sector in Europe. It can be reused for comparison against frameworks, other than CSEC**^+^**, to assess the need of education in specific cybersecurity sectors such as human security. Furthermore, the Qualtrics survey template (included) is a ready-made solution for replication studies.

**Specifications Table**

**Table:** 

Subject	Computer Science: Cryptography and Cybersecurity
Specific subject area	Assessment of cybersecurity skills in connection with job profiles, skill categories and significance of previous in the perspective of industry and academia respondents.
Type of data	Table Chart Figure
How the data were acquired	Data was acquired via surveys, where respondents selected the relevance of cybersecurity skills to perform in jobs. There were three phases: 1) worksheets distributed and collected individually via email; 2) online broadcast of worksheets, then uploaded by each respondent; 3) online broadcast with direct online input without worksheets. Data input by respondents consisted of numeric values from a Likert scale, indicative of the relevance of each of 55 skills (from the CSEC curricula of the ACM [1]) to perform in each of 6 job profiles relevant to cybersecurity. Also, the respondent's organisation type and country of origin were collected.
Data format	Raw Analyzed Filtered
Description of data collection	We selected six cybersecurity job profiles as a basis for the assessment that represented the border control scenario adequately and were generated in collaboration with project partners. The 55 cybersecurity skills used in our CSEC+ framework have been adapted from the ACM CSEC curricula [1]. No data normalization was required. The data was collected from survey respondents in Europe using existing project and professional networks.
Data source location	*Legend: Country (number of respondents)* Czech Republic (1); Denmark (5); Estonia (2); Finland (8); Germany (8); Greece (2); Hungary (2); Italy (8); Netherlands (13); Norway (1); Portugal (1); Slovenia (4); Spain (3); Sweden (2).
Data accessibility	*Repository name:* Figshare *Data identification number:* 10.6084/m9.figshare.22219573 *Direct URL to data:*https://figshare.com/articles/dataset/Consolidating_cybersecurity_in_Europe_A_case_study_on_job_profiles_assessment_data_package_/22219573
Related research article	Carlos E. Budde, Anni Karinsalo, Silvia Vidor, Jarno Salonen, Fabio Massacci, Consolidating cybersecurity in Europe: A case study on job profiles assessment, Computers & Security,Volume 127, 2023, 103082, ISSN 0167-4048, https://doi.org/10.1016/j.cose.2022.103082.

## Value of the Data

• The data enables the selection of key skills in the area of cybersecurity education. In addition, it displays the significance of specific cybersecurity skills according to industrial perspectives (as “consumers of education”) and academic ones (“producers of education”).
• All organisations and institutions with the objective of designing and implementing cybersecurity curricula for professionals, or which have a need for such cybersecurity skills (e.g. new workforce or training of existing professionals), can benefit from this data. Moreover, researchers can find this data useful as basis for their own work e.g. in case they wish to conduct similar assessments and comparisons.
• The data can be reused by conducting similar or parallel surveys. This can be done by choosing either similar or divergent job profiles compared to those assessed by this study. The study can also be replicated in different geographic areas, e.g. to compare the perception of cybersecurity skill needs across nations.

## 1 Objective

The dataset was collected in the Cyber Security for Europe project (funded by the European Union under the H2020 Programme GA#830929) study focusing on the skills required in distinct cybersecurity job profiles [2]. The context benefits all organisations and institutions having the objective of designing and implementing cybersecurity curricula for professionals or need such skills (e.g. new workforce or training of existing professionals).

The data has been used to report differences in priorities between academic organisations (“producers of education” for cybersecurity curricula) and industrial ones (“consumers of education”) when hiring new cybersecurity professionals and planning their training. The data was a significant part of the original research article mentioned in the “Related research article” section above.

## 2 Data Description

This dataset contains expert assessments of the cybersecurity skills required for six job profiles in Europe, as determined via surveys responded by cybersecurity experts from academia and industry. The data can be used to identify educational needs in the cybersecurity sector and compare against other frameworks.

The dataset includes tables with the survey responses (formatted as spreadsheet files), a bar chart (for statistical comparisons of responses between academic and industrial respondents), and a visualisation of overall results as a heatmap (a figure).

Brief descriptions of each file in the dataset, and its relationship to the main research article, are given in Table 1. Below we provide a more detailed description of the tables, chart, and figure.

**Table 1:** Description of files included in the dataset.

Filename	Description	*Related tables and figures in [**2**]*

survey_all_phases_summary.ods	Processed data of all respondents to the surveys in the article, including phases 1, 2, and 3.	*Tables 2 through 7, and table A.1 (Appendix A)*
survey_phase1_and _phase2.ods	Responses to phases 1 and 2 of the survey, as provided by the respondents, merged into a single spreadsheet.	*Ibid*
survey_phase3.ods	Responses to phase 3 of the survey: raw data provided by the Qualtrics online tool (https://vuass.eu.qualtrics.com/), and post-processing by the authors in separate sheets.	*Ibid*
survey_phase3.qsf	Qualtrics survey template, phase 3, to be visualised in www.qualtrics.com—free accounts can be created for this.	*Ibid*
barschart_areas_ACA_vs_IND.csv	Summary data from surveys, to compare the assessment of areas between academic and industrial respondents.	*Figure 6*
barschart_areas_ACA_vs_IND.gp	gnuplot script (http://www.gnuplot.info/) to convert the homonymous CSV data into a bar chart.	*Ibid*
barschart_areas_ACA_vs_IND.pdf	Result of executing the homonymous gnuplot script on the homonymous CSV data.	*Ibid*
heatmap.svg	Inkscape-generated SVG image (https://inkscape.org/) of the heatmap, based on the overall results of the surveys (columns F and G in survey_all_phases_summary.ods)	*Figure B.1 (Appendix B)*
heatmap.pdf	PDF version of heatmap.svg	*Ibid*

*Note on instructions for accessing these data:* data is provided in free and open formats (ASCII text files and scripts, OpenDocument Spreadsheets, PDF images and text files, scalable vectors graphics files), that can be visualised/modified/executed with freely available open software tools (e.g. Vim, gnuplot, LibreOffice, MuPDF, and Inkscape). The sole exception is the template survey_phase3.qsf—for the third-phase survey—that is to be open in the Qualtrics platform in www.qualtrics.com, which requires the creation of an account (free of charge). To facilitate the visualisation of that survey to people who do not want to create a free account in Qualtrics, we provide the link to the original survey: https://bit.ly/3QPHKXm. This permits public anonymous navigation of the third-phase survey.

**Tables**

The main content of this dataset is provided in spreadsheets, that record the raw results gathered in the surveys, and also include the filtering and post-processing phases used to produce the final results presented in the related research article [2]. There are three such spreadsheets:

• survey_phase1_and_phase2.ods contains the raw results of phases 1 and 2 of the survey—see Experimental design, materials and methods below.
  ○ Sheet “INSTRUCTIONS” contains the instructions provided to respondents.
  ○ Sheets “AC1” through “AC’14” contain each a full CSEC**^+^** framework expert assessment, accounting for the 13 responses gathered during the first survey phase.
  ○ Sheets “AC15” through “IND6” contain each a full CSEC**^+^** framework expert assessment, accounting for the 15 responses gathered during the second phase.
  ○ Further sheets therein with statistical information (averages and standard deviations of assessments) were used for preliminary conclusions, but were not used for data computations that appear in the final related research article.
• survey_phase3.ods contains the raw, filtered, and processed results of phase 3 survey:
  ○ Sheet “raw_data” contains the data extracted from Qualtrics—see Experimental design, materials and methods—representing the direct input provided by respondents, colour-formatted in the spreadsheet to ease visual interpretation.
  ○ Sheet “ready_data” contains a filter over the raw data, that discards invalid responses and compresses the useful information.
  ○ Sheet “summary” contains the CSEC**^+^**-framework-like result obtained from formatting the ready data (each row is a response), partitioned per job profile.
• survey_all_phases_summary.ods contains the final, processed data extracted from all valid responses gathered from the two files previously mentioned.
  ○ Sheets “Cybersec_Analyst” through “General_Cybersec_Auditor” contain each all responses gathered for the corresponding job profile, across all survey phases. The phases are divided by a black line, e.g. for the job profile “Cybersec_Analyst” rows 3–14 come from responses in phase 1, rows 15–29 come from phase 2, and rows 30–38 come from phase 3. In all these sheets, the first column (“Sector”) identifies whether the respondent comes from the academic sector, industrial sector, or other (e.g. public administration)
  ○ **Sheet “Summary” was used directly to produce the data presented in related research article**. It contains the average and standard deviation of each data point (i.e. a CSEC**^+^** skill for a job profile), as well as across all job profiles (columns “All: skill”) and aggregated per CSEC**^+^** area (columns “All: area”). The formulae used to compute this statistical information is embedded in the spreadsheet, viz. each cell contains the formula used to compute the value shown.
    To the right of this data, columns under “Demographics” (columns U through Z in the sheet) summarise the metadata of each collection phase, i.e. the type of organisation of the respondents and their country of origin. Also there is the computation of the confidence intervals used for the bar chart—see “Chart” below.
  ○ Sheets “Summary_ACA” through “Summary_managerial” are partitions of the sheet “Summary”, based on the origin of the responses (academic/industrial) or the type of job profile assessed (technical/managerial). **These sheets were also used directly for the data presented in the related research article**.

**Chart**

Besides general statistical information, we computed the statistical significance of the difference in *the average area value*^1^ between respondents from industry and academia. The goal was to determine, when the average value chosen (for an area) by industrial respondents differs substantially from those chosen by academic respondents, whether this difference is statistically significant. This was done by building confidence intervals using student's *T*-distribution with 59 degrees of freedom (considering that there are 60 independent data inputs, and using the standard rule of selecting n−1 degrees of freedom when there are n observations), and visually comparing their overlapping. The results are shown as bar plots in the file barschart_areas_ACA_vs_IND.pdf, computed using the included gnuplot script barschart_areas_ACA_vs_IND.gp on the resulting data that is provided as the CSV file barschart_areas_ACA_vs_IND.csv. Whiskers on top of the bars are 95%-confidence intervals.

**Figure**

Finally, a graphical representation was created for the general results of all three phases of data collection, i.e. the mean and standard deviation values that are in columns F and G of sheet “Summary” in file survey_all_phases_summary.ods. The visualisation aggregates the 60 assessments into one 6×55 transposed matrix, represented as a colour-blind-friendly heatmap. In that matrix, cell (i,j) contains a green circle that represents the mean and standard deviation of the 60 assessments for that i-th job profile and j-th skill:

- the mean value is represented by the lightness of the colour, where the darkest green stands for an assessment of 3 (i.e. advanced knowledge of skill j is required for job i), and the lightest green stands for an assessment of 0 (skill is irrelevant for the job);
- the standard deviation is represented by the diffusion of the circle borders, from a well-defined circle (corresponding to a standard deviation of 0.60 in the data) to a very-diffused circle (standard deviation of 1.20 in the data).

This visualisation allows readers to spot at a glance the hot (dark, signifying high skill needed) and cold (light, little skill needed) points of the whole study. This shows clearly the skills which are highly relevant to specific job profiles, in manner much easier to spot than looking at values in a table. The heatmap aggregates this information with a second dimension: the degree of consensus of the assessments, determined by the diffusion of the circle borders.

The image is provided in two formats: file heatmap.pdf is the portable version intended for easy visualisation by any party; file heatmap.svg is the scalable vectors graphics file used to produce that PDF.

## 3 Experimental Design, Materials and Methods

The data to collect consisted on expert assessments about the cybersecurity skills^2^ needed to perform in six job profiles, which had been selected based on their relevance for the cybersecurity-competence initiatives by the EC. These jobs are: (a) *General cybersec auditor*; (b) *Technical cybersec auditor*; (c) *Threat modelling engineer*; (d) *Security engineer*; (e) *Enterprise cybersecurity practitioner*; (f) *Cybersecurity analyst* 3.

Data input, which is a survey process to gather expert assessments, was standardised using the CSEC**^+^** framework: a cybersecurity skills framework that categorises 55 cybersecurity skills (“knowledge units” in the terminology of CSEC**^+^**) into nine “knowledge areas” as follows:

1. **Data security:**
  1.1 Cryptography
  1.2 Digital forensics
  1.3 Data integrity and authentication
  1.4 Access control
  1.5 Secure communication protocols
  1.6 Cryptanalysis
  1.7 Data privacy
  1.8 Information storage security
2. **Software security:**
  2.1 Fundamental principles
  2.2 Design
  2.3 Implementation
  2.4 Analysis and testing
  2.5 Deployment and maintenance
  2.6 Documentation
  2.7 Ethics
3. **Component security:**
  3.1 Component design
  3.2 Component procurement
  3.3 Component testing
  3.4 Component reverse engineering
4. **Connection security:**
  4.1 Physical media
  4.2 Physical interfaces and connectors
  4.3 Hardware architecture
  4.4 Distributed systems architecture
  4.5 Network architecture
  4.6 Network implementations
  4.7 Network services
  4.8 Network defence
5. **System security:**
  5.1 System thinking
  5.2 System management
  5.3 System access
  5.4 System control
  5.5 System retirement
  5.6 System testing
  5.7 Common system architectures
6. **Human security:**
  6.1 Identity management
  6.2 Social engineering
  6.3 Personal compliance with cybersecurity rules/policy/ethical norms
  6.4 Awareness and understanding
  6.5 Social and behavioural privacy
  6.6 Personal data privacy and security
  6.7 Usable security and privacy
7. **Organisational security:**
  7.1 Risk management
  7.2 Security governance and policy
  7.3 Analytical tools
  7.4 Systems administration
  7.5 Cybersecurity planning
  7.6 Business continuity, disaster recovery, and incident management
  7.7 Security program management
  7.8 Personnel security
  7.9 Security operations
8. **Societal security:**
  8.1 Cybercrime
  8.2 Cyber law
  8.3 Cyber policy
  8.4 Privacy
9. **Operate and maintain:**
  9.1 Customer service and technical support

The CSEC**^+^** framework also defines a 0–3 Likert scale on the relevance of a skill for a job:

1. **Irrelevant**: the skill or knowledge is not necessary to perform in the given job specialization.
2. **Basic knowledge**: understanding the basic principles of the skill or knowledge is needed in the job specialization; application of these is not necessary to perform in the specialisation.
3. **Intermediate knowledge**: applying the skill or knowledge is needed to perform in the specialization; application is only needed up to the point of well-known standard procedures.
4. **Advanced knowledge**: applying the skill or knowledge is essential to perform in the specialization; the application of the skill or knowledge is necessary on an advanced level and beyond well-defined standard procedures.

Thus, data input by an assessor for a job profile consists on the following steps:

(a) Select one of the six cybersecurity job profiles (a)–(f) listed above;
(b) Select one of the nine knowledge areas from CSEC**^+^**;
(c) For each skill in the area, indicate on the 0–3 Likert scale its relevance to perform in the job profile that was selected in item a);
(d) Repeat items b) and c) for a different knowledge area, until all areas have been covered.

The result of such input is a 6×55 matrix of values in the integral range [0,3], where a row represents a job profile (six in total), and a column represents a skill from a knowledge area (55 in total). In other words, the data cell value (i,j)∈{0,1,2,3} of the matrix stands for the relevance of the j-th skill to perform in the i-th job profile according to the respondent who filled the matrix.

*Such matrix is called “an expert assessment using the CSEC****^+^****framework”*.

**Fig. 1:**
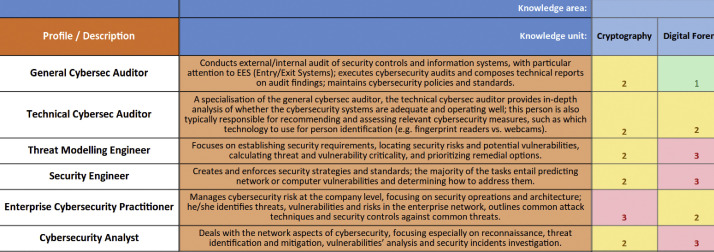
job descriptions (second column) embedded in the assessment matrix of a survey.
This phase gathered 13 responses from partners in Finland, Italy, Slovenia, and Denmark.
  c. The corresponding expert assessments are in sheets AC1 through AC’14 in the spreadsheet file survey_phase1_and_phase2.ods. Sheet AC’8 contains an almost empty matrix, and it is included solely for completeness and to indicate that no assessment was provided by that respondent.
(2) **The second phase** (Mar–Apr 2022) was an online service where the survey could be downloaded, filled in, and uploaded. Dissemination was broadened to include ECSO^4^, and the CS4E-sibling projects CONCORDIA, Sparta, and ECHO. The online service allowed for anonymous input. This is in contrast to phase 1, where data had been anonymised after aggregating the assessments. Therefore, respondents provided metadata in the online form.
  a. For metadata, respondents were asked the type of organisation in which they work (*Large company, SME, Academic/Research, Public administration, Other*) as well as their country of origin.
  b. Data input was performed as in phase 1: even though the distribution of the assessment matrix was anonymous and online, respondents still downloaded, filled in, and uploaded the matrix as in phase 1. The instructions and job profiles are thus also included in the spreadsheet file survey_phase1_and_phase2.ods—see Fig. 1.
This phase gathered 15 responses from the Czech Republic, Estonia, Germany, Greece, Hungary, Italy, Norway, and Spain.
  c. The corresponding expert assessments are in sheets AC15 through IND6 of the spreadsheet file survey_phase1_and_phase2.ods. Sheets AC’18 and AC’19 are a duplicate: their data is identical, and it was interpreted only once.
(3) **The third phase** (Sep–Oct 2022) was an online survey prepared with Qualtrics, allowing for responses to be input directly without file exchanges. It was deployed for PC and mobile devices, using dissemination via personal communication, mailing lists, and the CS4E public newsfeed—see Fig. 2 and the page in the official CS4E website^5^.
  a. All input was anonymous and online, data and metadata, which includes the instructions on how to perform the assessment using the CSEC**^+^** framework.
  b. The survey per se is included as file survey_phase3.qsf, to be loaded in the Qualtrics platform. Alternatively, since it will be kept online (but no further input data will be processed), the survey experience can be replicated by visiting https://bit.ly/3QPHKXm.

The expert assessments described above were gathered via surveys disseminated in the cybersecurity community. More in detail, data collection was divided in three phases:

(1) **The first phase** (Oct 2021–Jan 2022) collected initial data and served to refine a larger-scale process. Dissemination, targeted at CyberSec4Europe partners, consisted in intra-CS4E announcements and subsequent communication via private E-mails, which contained the survey with instructions about the CSEC**^+^** framework and the job profiles to assess.
  a. Disseminated instructions are in the first sheet (“INSTRUCTIONS”) of the spreadsheet file survey_phase1_and_phase2.ods of this dataset.
  b. Descriptions of the job profiles assessed using the CSEC+ framework are included in the same file, embedded in the assessment matrix between the job titles and the assessment—see Fig. 1.

**Fig. 2:**
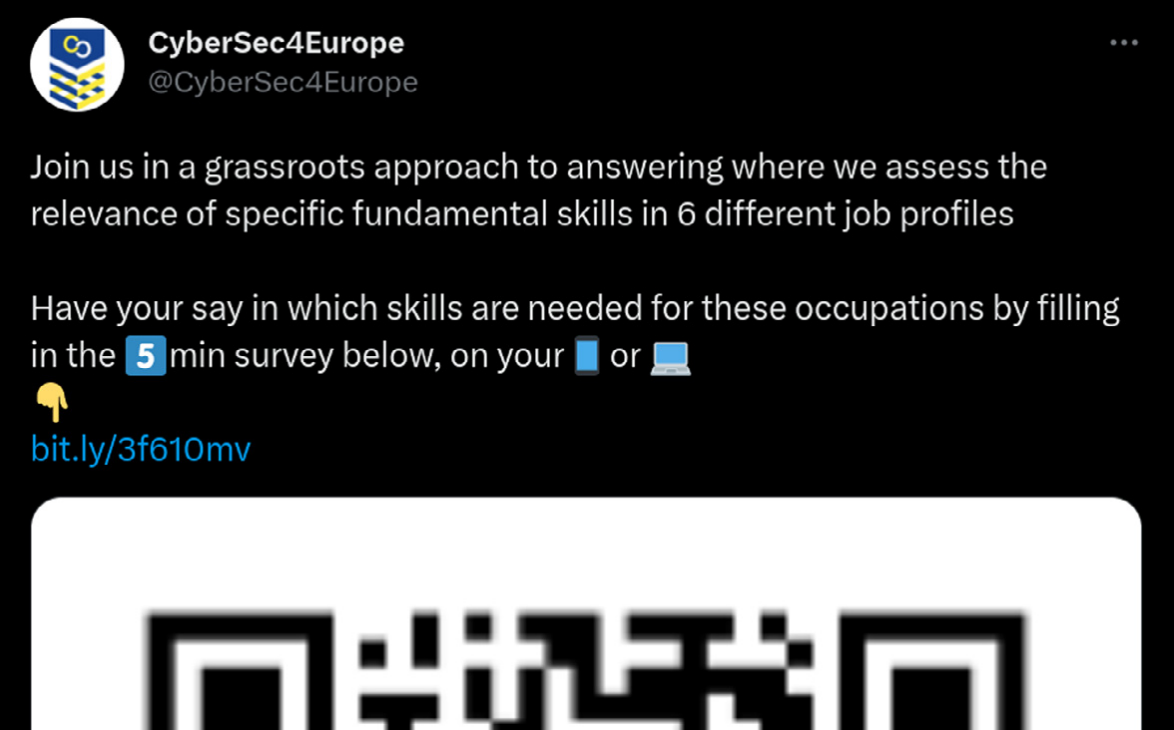
survey phase 3 publicised in the Tweet account of CS4E^6^.
This phase gathered 32 responses from Denmark, Estonia, Finland, Germany, Greece, Italy, The Netherlands, Portugal, Slovenia, and Sweden.
  c. The corresponding data extracted from the Qualtrics platform is located in sheet “raw_data” of the spreadsheet file survey_phase3.ods. Sheets “ready_data” and “summary” in the same file are filters and restructurings of data representation from that raw (uncleaned) data, performed to ease a later analysis.

Statistical analyses were applied to the collected data to compute mean values and standard deviations for different data partitions, all included in file survey_all_phases_summary.ods:

- The curated data, which removes invalid entries, and the metadata (assessors’ countries and organisation types) for all survey phases are summarised in the “Summary” sheet of that file.
- Assessments by industrial respondents (assessors that chose “Large company”, “SME”, or “Public administration” in the metadata) were compared against assessments by academic respondents (assessors that chose “Academic/Research”)—see sheets “Summary_ACA” and “Summary_IND” in that file.
- Responses for managerial job profiles (Enterprise Cybersec Practitioner, General Cybersec Auditor, Technical Cybersec Auditor) were compared against responses for technical job profiles (Cybersec Analyst, Security Engineer, Threat Modelling Engineer)—see sheets “Summary_managerial” and “Summary_technical” in that file.

The difference in the average area value—i.e. the average values chosen for all skills pertaining to a knowledge area—between assessors from industry and academia was also computed. This was done by building confidence intervals using student's *T*-distribution with 59 degrees of freedom (see section Data description: Chart). The results are shown as bar plots in the file barschart_areas_ACA_vs_IND.pdf, computed using the included gnuplot script barschart_areas_ACA_vs_IND.gp on the resulting data that is provided as the CSV file barschart_areas_ACA_vs_IND.csv. Whiskers on top of the bars stand for the width of the confidence intervals.

Finally, a graphical representation was created for the global statistical results, i.e. the mean and standard deviation values that are in columns F and G of sheet “Summary” in file survey_all_phases_summary.ods. The visualisation was created manually using Inskape for SVG edition. Color-coding was designed to be colour-blind-friendly—details with RGB coordinates and their perception by different types of colour blindness is provided in the file heatmap.svg: the “Green palette” was chosen over the “Blue–Red palette” by expert advice.

## Ethics Statements

This work gathered the textual input of human subjects in a standardised format, namely the CSEC**^+^** framework described above. Respondents would provide input after reading an informed consent statement that described the purpose, use, and dissemination of results of the study, and identified participation in the study as anonymous and voluntary. The resulting input is a numeric assessment in the integral range [0,3] of skills mapped to job profiles, that cannot be reasonably expected to be usable for the identification of respondents.

Furthermore, all collected input was anonymised before uploading files to Figshare at DOI 10.6084/m9.figshare.22219573. This includes all metadata other than two demographic questions used in the study (country of origin and type of organisation of the survey respondent).

This work did not involve any other subjects, experiments or ethically questionable topics.

## CRediT authorship contribution statement

**Carlos E. Budde:** Conceptualization, Methodology, Validation, Investigation, Data curation, Writing – original draft, Writing – review & editing, Visualization, Supervision, Funding acquisition. **Anni Karinsalo:** Conceptualization, Validation, Investigation, Data curation, Writing – original draft. **Silvia Vidor:** Validation, Investigation, Writing – original draft, Visualization. **Jarno Salonen:** Validation, Project administration, Funding acquisition. **Fabio Massacci:** Methodology, Validation, Writing – review & editing, Supervision, Project administration, Funding acquisition.

## Declaration of Competing Interest

The authors declare that they have no known competing financial interests or personal relationships that could have appeared to influence the work reported in this paper.

## Data Availability

CSEC+ Framework Assessment Dataset: Expert Evaluations of Cybersecurity Skills for Job Profiles in Europe (data package) (Original data) (Figshare).

## Footnotes

6 https://twitter.com/CyberSec4Europe/status/1572215360916541444?ref_src=twsrc%5Etfw.

1 The average of the values chosen for all skills pertaining to a knowledge area of CSEC**^+^**.

2 All raw collected data is in the spreadsheet files with “.ods” extension—see Table 1.

3 Job profiles selection is described in § 3.1 of the D6.6 deliverable from the H2020 project CS4E.

## Footnotes

4 The European Cyber Security Organisation (ECSO: https://ecs-org.eu/) is a European organisation for the development of cybersecurity communities and the European cybersecurity ecosystem.

5 https://cybersec4europe.eu/the-eu-cybersecurity-skill-gap-is-bleeding-but-you-can-help-heal-it/.
